# Teenage Obstetrics in Bristol

**Published:** 1964-10

**Authors:** M. B. Wingate

**Affiliations:** University Department of Obstetrics and Gynaecology, Bristol


### TEENAGE OBSTETRICS IN BRISTOL

BY

M. B. WINGATE

*University Department of Obstetrics and Gynaecology, Bristol*

An increasing proportion of obstetric practice is now concerned with the ante-natal care and delivery of the teenage mother, and if sociological reports are correct this proportion will continue to increase. Recent papers on pregnancy and labour in this group of women have emphasized the higher incidence of anaemia, pre-eclampsia, premature and prolonged labour and puerperal pyrexia, when compared with older primigravidae. Additionally, cephalo-pelvic disproportion was said to occur commonly and to be responsible for a high operative delivery rate.[1, 4, 6, 8, 9, 10] Some of the papers dealt with American patients in widely varying social classes, and as has been pointed out by Baird,[2] this fact must be taken into account when analysing obstetric performance.

Because a contrary impression was gained in looking after teenage mothers in Bristol, it was decided to review the first pregnancy occurring in girls aged sixteen and under during the five year period 1957 to 1962 in the Bristol Hospitals Clinical area. It must be emphasized that the findings relate to hospital delivery only.

### MATERIAL AND METHODS

The majority of hospital confinements in the area are dealt with in A and B blocks of Southmead Hospital and in the Bristol Maternity Hospital, and teenage mothers having their first confinements are given priority bookings.

The information for this study was obtained from the case records of both hospitals from 1st January 1957 to 31st December 1961. A total of 274 teenage patients between the ages of thirteen and sixteen years were delivered in the two hospitals concerned.

A control group of 589 primigravid patients aged 17 years and above, who had been delivered in A block of Southmead hospital during the year 1960, was used as a measure of the incidence of the various complications discussed.

The complications and their incidence have been set out in table form, side by side with the figures for the control group.

TABLE I

Complications of Pregnancy

| | Teenagers | Controls |
|---|---|---|
| Anaemia | 18 ( 6·5 per cent) | 58 (10 per cent) |
| Pre-Eclampsia | 52 (18·9 per cent) | 167 (28·3 per cent) |
| Premature Labour | 39 (10·8 per cent) | No relevant figures |
| Post-Maturity | 36 (13·1 per cent) | No relevant figures |
| Multiple Pregnancy | 2 ( 0·7 per cent) | 10 (1·6 per cent) |

### THE ANTE-NATAL PERIOD

All unmarried mothers are routinely investigated for evidence of venereal disease and the majority of teenagers were unmarried at the time of booking at an ante-natal clinic. It was surprising to find that only two girls had gonorrhoea and one syphilis.

Several reports on teenage pregnancy describe a high incidence of anaemia.[6, 8, 10] In this series a patient was considered to be anaemic if the haemoglobin level fell below 75 per cent (11·1 g) during the pregnancy. From the figures given in Table I it cannot be considered that anaemia is a more prominent finding in teenage pregnancy.

There was complete agreement in all the reports studied on the frequency with which teenage pregnancy was complicated by pre-eclampsia, the rate often being quoted as three or four times higher than the average for the older primigravid population. Opinions differ as to the cause, but inadequate diet,[10] lack of ante-natal care,[3] under-development of the endocrine system,[8] and stress,[7] have been suggested. A diagnosis of pre-eclampsia was made if two or more of the following features were present (1) blood pressure above 130/80, (2) oedema of the hands or feet, (3) albuminuria not due to other causes, (4) excessive weight gain. Because of interest in the effects of stress on the incidence of pre-eclampsia, the figures obtained were divided into unmarried and married teenagers. Of the total of 52 (18·9 per cent) of teenage patients who developed pre-eclampsia during their pregnancies 17 (6·3 per cent) were married and 35 (12·6 per cent) were single.

Although the incidence of pre-eclampsia is higher amongst controls than amongst teenagers, it must be remembered that some of the older primigravidae were referred to the Unit for delivery because they developed this complication. Comparison is not, therefore, strictly valid.

The majority of premature labours, that is labour beginning before the 38th week of pregnancy, were induced for such conditions as pre-eclampsia and hypertension; and because management of these conditions differs in the two hospitals concerned, no comparative control figures can be given. The same difficulty arose in considering pregnancies prolonged beyond forty-two weeks, but the figures for teenage pregnancies are given as they may be of interest.

Complicating medical conditions were rare amongst the teenage group, one patient having heart disease and one diabetes mellitus.

It was of interest to note that at the time of their delivery 190 of the girls were unmarried and 84 were married.

### LABOUR AND DELIVERY

Most authors report no essential difference in the duration of labour of teenage and older primigravid mothers, and this is borne out in the Bristol survey. In 271 of the 274 teenage labour records, figures were available for the duration of the first, second and third stages of labour. These were separately totalled and averaged. The average duration of the first stage was 11·38 hours, of the second stage 38·7 minutes and of the third stage 9·3 minutes.

TABLE II

Complications of Labour

| | Teenagers | Controls |
|---|---|---|
| Breech Deliveries | 18 ( 6·5 per cent) | 22 ( 3·7 per cent) |
| Prolonged Labour | 23 ( 8·7 per cent) | 22 ( 3·7 per cent) |
| Forceps Delivery | 35 (12·1 per cent) | 74 (12·5 per cent) |
| Caesarean Section | 3 ( 1·1 per cent) | 19 ( 3·2 per cent) |
| Post-partum Haemorrhage | 17 ( 6·2 per cent) | 32 ( 5·4 per cent) |
| Manual Removal of Placenta | 8 ( 2·8 per cent) | 13 ( 2·3 per cent) |
| Puerperal Pyrexia | 21 ( 7·8 per cent) | 15 ( 2·5 per cent) |

No difference was noted in the method of delivery between the teenage and control group. The incidence of forceps delivery was the same for both groups, while the incidence of Caesarean section was lower in teenage than in the older primigravida group of mothers. In the papers reviewed, most authors stress the high Caesarean section rate in the delivery of teenage patients, in the main for cephalo-pelvic disproportion. There were only two teenage patients with clinically and radiologically proven disproportion and one had a successful vaginal delivery. These findings together with the high average birth weight of their babies suggest that disproportion is not a problem in teenage obstetric practice.

The incidence of postpartum haemorrhage and the need for manual removal of the placenta are the same for both groups studied.

An oral temperature of 100·4°F or more occurring at any time during the postpartum hospital stay was used as a criterion for morbidity. There was a considerably higher incidence of puerperal pyrexia amongst the teenage mothers than amongst the controls.

There were no maternal deaths in either the control or study group during pregnancy, delivery, or the post-partum period.

### THE BABY

The weights of all babies born to the teenage group were totalled and averaged. Including the two sets of twins the average baby weight was 7·06 pounds, the smallest weighing 2 pounds 11 ounces and the largest 11 pounds. Eleven babies weighed under $5\frac{1}{2}$ pounds, including the two sets of twins, an incidence of 2·1 per cent. This percentage of premature babies is the same as that of the control group and is the average quoted in most studies.

Six infants were found to have congenital abnormalities, an incidence of 2·1 per cent; the incidence is the same for the control group.

There were four perinatal deaths amongst the babies born to the teenage group, an incidence of only 14 per thousand. This figure is low when compared with the national average and with the control group where the perinatal mortality was 27 per thousand.

### SUMMARY

A comparison has been made between pregnancy and labour in 274 teenage mothers, delivered in hospitals in the Bristol Clinical area from 1957 to 1962, and a control group of 589 older primigravidae.

No essential difference between the two groups was noted in respect of the incidence of malpresentation, forceps delivery, Caesarean section, postpartum haemorrhage, or manual removal of the placenta; and anaemia and pre-eclampsia were less common amongst teenage patients in this study.

The average duration of labour in teenage mothers is by no means excessive, although there is a greater incidence of prolonged labour and of puerperal pyrexia.

The babies of teenage mothers are healthy, of good average weight, and have a higher survival rate than those of older and more mature primigravidae.

This study emphasizes the fact that pregnancy in the teenage patient presents no greater difficulties than obstetrics in general.

*Acknowledgements*

I wish to thank the consultant staff of the Bristol Maternity Hospital and B Block of Southmead Hospital for permission to use their case records.

### REFERENCES

1. Arnot, P. H. and Nelson, D. R., *West. J. Surg.* (1958), **66,** 332.
2. Baird, D., Hytten, F. E. and Thomson, H. M., *J. Obst. and Gynaec. Brit. Emp.* (1958), **65,** 865.
3. Clark, J. F., McDaniel, J. B. and Cherrie, E. E., *J. Nat. M.A.* (1962), **54,** 352.
4. Harris, J. W., *Bull. Johns Hopkins Hosp.* (1922), **33,** 12.
5. Hulka, J. F. and Schaaf, J. T., *Obst. and Gynaec.* (1964), **23,** 5, 678.
6. Israel, L. S. and Wautersz, T. B., *Am. J. Obst. and Gynec.* (1963), **85,** 659.
7. Lennon, G. G., Personal Communication.
8. Marchetti, A. A. and Menaker, J. S., *Am. J. Obst. and Gynec.* (1950) **59,** 1013.
9. Salzmann, B., Keating, W. S., and Head, T., *Current M. Digest* (1954), **24,** 108.
10. Semmens, J. D. and McGlanary, J. C., *Obst. and Gynaec.* (1960) **16,** 31.

